# Editorial: Mindfulness, mind-body exercises, and health promotion

**DOI:** 10.3389/fpsyg.2025.1559535

**Published:** 2025-02-04

**Authors:** Guodong Zhang, Yang Cao, Zi Yan

**Affiliations:** ^1^College of Physical Education, Institute of Sport Science, Southwest University, Chongqing, China; ^2^Clinical Epidemiology and Biostatistics, School of Medical Sciences, Faculty of Medicine and Health, Örebro University, Örebro, Sweden; ^3^School of Health Sciences, Merrimack College, North Andover, MA, United States

**Keywords:** mind-body exercise, mindfulness, Daoism, stillness, restoration, health promotion (HP)

## Introduction

Physical activity provides numerous benefits for both physical and mental health, such as improving physical fitness, promoting cardiovascular health, and enhancing muscle strength (Qiu et al., 2023). In particular, mind-body exercises rooted in Eastern traditional cultures, such as Tai Chi, Qigong, and Yoga, have demonstrated unique advantages in promoting mental health and physical recovery (Deuel and Seeberger, 2020; Lin et al., 2019). These practices emphasize mind-body unity and seek to achieve balance through slow and deliberate movements, deep breathing, and meditation (Kung et al., 2024). Recent studies highlight the significant advantages of mind-body exercises on physiological health, mental wellbeing, and nervous system function (Dong et al., 2024; Loewenthal et al., 2023; Tao et al., 2019). Despite this growing body of evidence, the specific mechanisms through which these exercises influence physical and mental health recovery remain unclear. This Research Topic comprises a series of articles aimed at elucidating the health benefits of mind-body exercises from the perspectives of psychological benefits, physiological effects, and emerging technologies, in order to provide a more comprehensive assessment of their impact and potential. The topic explores the health-promoting effects and mechanisms of mind-body exercises from three perspectives: psychological benefits, physiological effects, and technological approaches.

## Psychological benefits

Seven studies emphasize the psychological benefits of mind-body exercises. Three cross-sectional studies, employing large-sample mediation models, confirmed the role of mind-body exercises in promoting mental health (Tang et al.; Wu et al.; Zhang et al.). Wu et al. found that practicing Tai Chi had a more pronounced impact on the mental health of elderly individuals living alone compared to Baduanjin and walking exercises, with social participation and exercise environment serving as mediating and moderating roles. Tang et al. discovered that mindfulness practice could improve state-trait anxiety and stress resilience in athletes prone to choking, with resilience and perceived stress acting as mediators. Zhang et al. found that self-compassion predicted greater emotional recovery following failure in athletes, with vagal nerve response mediating this effect.

Additionally, four studies focused on the psychological health benefits of yoga. Malipeddi et al. found that practicing Isha Yoga 3–4 times per week during the COVID-19 pandemic significantly reduced stress and mental distress while improving overall wellbeing. Chhajer and Dagar highlighted the dual benefits of yoga, noting that yoga training not only improved participants' thriving and overall health but also significantly alleviated psychological issues such as stress and anxiety. Nadholta et al. further confirmed that practicing yoga during pregnancy alleviated symptoms of stress, anxiety, and depression, while also reducing physical discomfort, fostering mind-body balance, and enhancing mother-infant relationships. Lastly, Yang et al. conducted a meta-analysis that validated the effectiveness of mindfulness-based yoga in treating depression.

These seven studies contribute significantly to the growing body of evidence supporting the mental health benefits of mind-body exercises and offer a robust theoretical foundation for refining and advancing methodologies in this field.

## Physiological benefits

One of the challenges in this field is expanding the scope of research on the physiological impacts of mind-body exercises to explore a greater diversity of health outcomes. Six studies underscore the physiological benefits of mind-body exercises. Wang et al. found that 8 weeks of Tai Chi practice enhanced bed rest time, total sleep duration, and stage 2 of non-rapid eye movement sleep in elderly individuals. Van de Winckel et al. reported that Qigong practice had positive effects on the rehabilitation and quality of life of patients with spinal cord injuries, including pain reduction, improved sleep, and greater emotional stability. Thakur et al. discovered that meditation practice increased telomerase activity and telomere length while lowering cortisol levels and improving mental health, suggesting its potential as a biomarker for combating human aging. Bartenschlager and Jansen found that meditation reduced defensive responses to death-related stimuli, improved emotional regulation, lowered stress and anxiety, and enhanced overall wellbeing.

In addition, two studies emphasized the dual benefits of mind-body exercises for both physiological and psychological health. Solk et al. found that integrating mindfulness meditation with exercise reduced participants' cognitive load during physical activity, making exercise feel easier, while improving emotional regulation and mental state. This led to greater enjoyment of the exercise process and increased self-efficacy. Gu et al. similarly found that dance-based exercise games significantly boosted enjoyment among college students and positively influenced energy expenditure and self-efficacy. These studies highlight the impact and mechanisms of mind-body exercises in promoting physiological health, providing valuable evidence for developing related exercise training programs.

## Technological approaches

The final area of this Research Topic explores the technological approaches, with a particular focus on Virtual Reality (VR) technology. Gao et al. provided the first comprehensive review of VR applications in meditation and teir impact on the physical and mental health of elderly individuals. The study found that VR meditation enhanced exercise quality by delivering real-time visual and auditory feedback. Additionally, the immersive VR experience made it easier for participants to engage in and enjoy meditation practice. However, the implementation of these interventions still faces challenges, such as high costs and issues like simulator sickness. Moreover, Mortlock et al. investigated how online mindfulness practices during the COVID-19 pandemic fostered trust, connection, and shared humanity within a community. Using interdependence theory, their study explored the social functions of shared mindfulness practices and proposed strategies for cultivating mindful communities, calling for further research to refine these practices across various settings.

## Concluding remarks

The benefits of mind-body exercises for both physiological and psychological health have been well-established in this topic. The roles of Tai Chi, yoga, and meditation in improving sleep quality, reducing anxiety and stress, enhancing emotional regulation, and increasing overall wellbeing have been thoroughly explored. Additionally, mind-body exercises demonstrate promise in combating aging and promoting overall health through mechanisms such as reducing cognitive load during exercise, extending telomere length, and increasing telomerase activity.

Future research should continue to explore the specific mechanisms by which mind-body exercises impact health, particularly in regulating psychological and physiological processes to achieve positive health outcomes. Researchers should also focus on how various populations, age groups, and health conditions respond to mind-body exercises to design more tailored and effective training programs.

In terms of technology, VR merits further investigation, though challenges such as cost and simulator sickness must be addressed. Moreover, greater emphasis should be placed on community-based mindfulness applications to foster trust, connection, and shared humanity. Through continued research and innovation, mind-body exercises have the potential to become a vital tool for improving physical and mental health, playing a key role in promoting longevity, alleviating disease symptoms, and enhancing quality of life.
